# The authors’ response

**DOI:** 10.1186/s40560-020-00493-w

**Published:** 2020-09-26

**Authors:** Ofer Sadan, Owen Samuels

**Affiliations:** Department of Neurology and Neurosurgery, Division of Neurocritical Care, Emory University Hospital, 1364 Clifton Rd. NE, Atlanta, GA 30322 USA

**Keywords:** Cerebral edema, Subarachnoid hemorrhage, Hyperosmolar therapy, Hyperchloremia, Acute kidney injury, Neurocritical care

## Abstract

In response to comments raised, we acknowledge the shortcomings of our study. It is a small study. However, it is a pilot study, which is not meant to create generalizable data, rather to explore new potential directions. To this end, our conclusions were clearly supported by the results. We demonstrated that administration of 16.4% NaCl/Na-acetate solution was feasible, safe, and was associated with lower rates of AKI. We share the call that large RCTs are required to follow this pilot study and hope that our data will stimulate the ongoing discussion regarding the role of chloride in AKI mechanism.

To the editor,

We appreciate the interest in our manuscript [1], and the opportunity to respond to the comments. There is no doubt that our study suffers from several shortcomings. It is a small study, and even smaller than planned due to limitations that were not always in our control. However, it is a pilot study, as the title mentioned. Pilot studies are not meant to create generalizable data, rather to explore new potential directions. We believe that our study accomplished that goal.

To our knowledge, our study is the first of its kind, presenting high-quality prospective data, which demonstrated the feasibility and potential safety of an alternative hypertonic solution. Although our study was underpowered to reach its primary endpoint, the results did point to our hypothesized direction. This phase 1-type research is the beginning, not the end of the clinical investigation, and should be viewed and our findings considered preliminary in nature.

The patients at our ICU are often treated according to sodium goals in order to improve intracranial compliance. Therefore, using less doses suggests that we were able to reach the goal more efficiently with the alternative solution, not that these patients had less edema. As for the delta creatinine, we reported that the majority of AKI events met the criteria of KDIGO 1, which means a small change in creatinine, or reduced urine output. Therefore, it is of no surprise that the change in creatinine was undetected in such a small group of patients.

We agree that there are likely additional factors that could have been considered in the analysis, such as vasopressor use, and perhaps specific nephrotoxins. However, when dealing with a small cohort, having too many competing variables will mask any effect, simply from a statistical-mathematical calculation standpoint. In a larger trial, additional factors will be considered.

The timing of AKI did surprise us. We designed the study in such a way that only patients at higher risk of hyperchloremia-associated AKI would be randomized. With this intent, patients were randomized only when mild hyperchloremia was measured. With the clarity of hindsight, it would have been better if we would have randomized patients earlier, since the temporal relationship between hyperchloremia and AKI was shorter than expected.

We would like to thank the comment regarding Fig. 3b. Indeed, this was a mistake. We did have 9 patients who developed AKI in the NaCl group yet presented only 8 in that graph. We apologize for this mistake, and an erratum with the corrected graph was published.

**Fig. 3:**
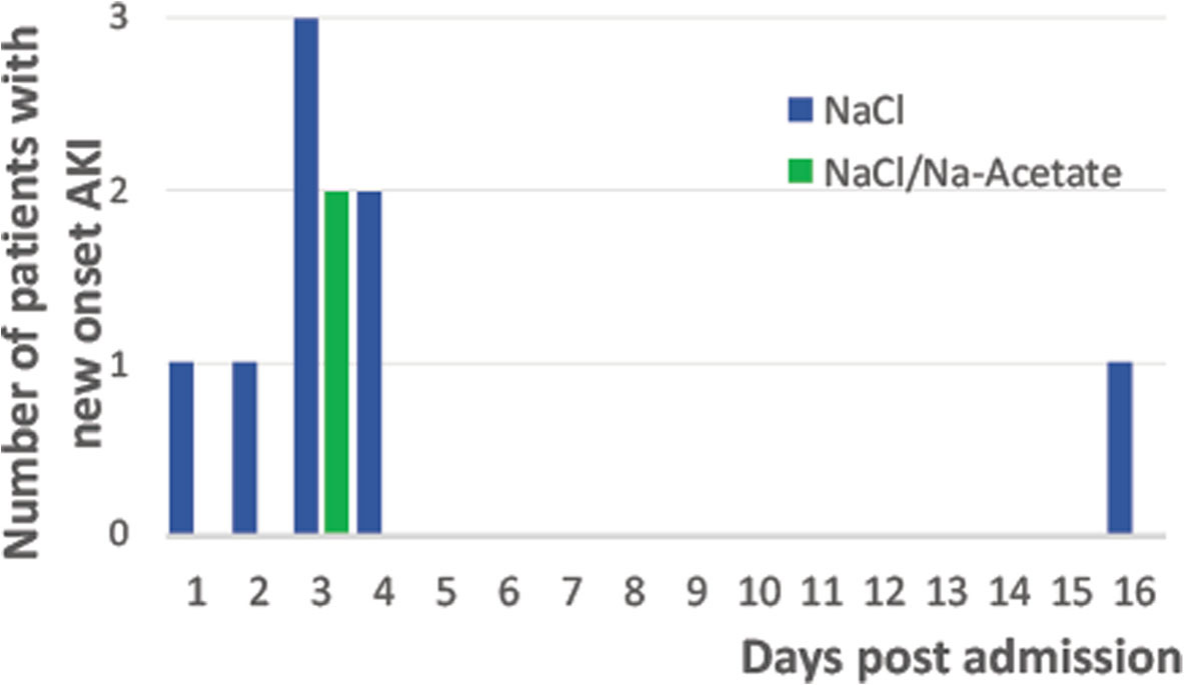
**b** Histogram of AKI frequency by group of treatment and hospitalization day

Overall, despite the intrinsic limitation of a small randomized pilot study, we believe our conclusions were clearly supported by the results. We demonstrated that administration of 16.4% NaCl/Na-acetate solution was feasible, safe, and was associated with lower rates of AKI. We share the call that large RCTs are required to follow this pilot study and hope that our data will stimulate the ongoing discussion regarding the role of chloride in AKI mechanism.

## Data Availability

The datasets used and/or analyzed during the current study are available from the corresponding author on reasonable request.

## Abbreviations

ICU: Intensive care unit
AKI: Acute kidney injury
